# MMC celebrating 6 years of experience and expansion

**DOI:** 10.1111/1753-0407.13270

**Published:** 2022-05-11

**Authors:** Itamar Raz

**Affiliations:** ^1^ Diabetes Medical Center Tel Aviv Israel

More than 530 million people are now affected by the global diabetes epidemic.^1^ Diabetes is particularly an issue in developing countries. By 2030, the *International Diabetes Federation (IDF) 2021 Atlas* predicts that the number of patients with diabetes will rise to 643 million, with three in four adults with diabetes living in low‐ and middle‐income countries.^1^ Of this large number of persons with diabetes in low‐ and middle‐income countries, more than 60% are in Asia and approximately one‐third in China. The latest epidemiological study in China shows that nearly 11% of the population is diagnosed with diabetes,^2^ leaving a large proportion undiagnosed yet, showing that China has experienced one of the most remarkable increases anywhere in the world.

If combined with diabetes, chronic diseases, such as chronic kidney disease, cardiovascular disease, stroke, infection, and some kinds of cancers, are associated with higher mortality.^2^ Diabetes‐related medical expenses in China have surged from 2.2 billion renminbi (RMB) to 200 billion RMB between 1993 and 2007 and by 2030 are expected to exceed 360 billion RMB. The challenge of diabetes management to China is considered to be one of the most important examples in the world, which has brought an enormous burden to the medical system.^2^

## CONTROL OF PRIMARY RISK FACTORS

In China, hypertension is common and its prevalence is rising. Among adults aged 35 to 75 years, the low control rate is so ubiquitous in all subgroups that a broad‐based global strategy is in great need, such as more prevention efforts, better screening, and cost‐effective treatment. Awareness, treatment, and control of hypertension and dyslipidemia in China are much lower than in the United States.^3^

People in China have a high prevalence of cardiovascular risk factors due to growth of the ageing population, lack of diabetes educators, and lack of structured health care delivery. Social isolation, social disparity, and unequal access to care, along with high prevalence of overweight and obesity and low socio‐economic status lead to a lack of awareness of diabetes.^4^

In order to improve the management of chronic disease in China, effective prevention of risk factors is essential. Based on the major predictive factors age, sex, waist circumference, body mass index, systolic blood pressure, and family history of diabetes, researchers developed a Chinese diabetes risk score to help identify high‐risk individuals.^5^ In view of the large number of high‐risk groups in China, comprehensive behavioral intervention to increase physical activity and improve diet is key to diabetes prevention efforts. Diabetes nurses, nutritionists, pharmacists, and doctors should be available and be more involved in patient care, both face to face and through digital medicine.^4^, ^6^

There are gaps between recommended guidelines and the real‐world health care for diabetes due to wide variations in economy, cultural development, and medical service standards in different regions in China. In seeking methods to address all these challenges, the National Metabolic Management Center (MMC) was founded in 2016 as a pilot and standard system that can be replicated throughout the country.^7^

MMC aims to launch a new community management model of metabolic diseases based on an Internet health information platform. In addition, MMC helps to improve patients' compliance and effectiveness of treatment, so as to benefit patients, doctors, and medical staff. The proprietary electronic medical database in MMC supports dynamic big data analysis on epidemiology, prevention, diagnosis, and treatment of diabetes. In addition, the nationwide standardized care system also provides a collaborative platform for prospective interventional studies on treatment and prevention of diabetes.^8^, ^9^, ^10^ Already, a large body of data and evidence originating from hospital‐based care in MMC clinics has helped to achieve long‐term optimal health outcomes,^4,7^ thus enabling a replicable standard care model across the country, laying the foundation for future medical policy formulation and improvement. The goal of MMC is to reduce the incidence rate of diabetes by 1% and the rate of diabetes‐related complications by 10% in China.

Artificial intelligence technology‐based medical tools were developed to promote screening, diagnosis, and treatment of diabetes in MMC clinics. In addition, the MMC is devoted to establishing highly efficient diagnosis and treatment, as well as comprehensive disease management, both in and outside of the hospital setting.

By the end of 2018, it was estimated that there would be over 300 MMC clinics and that their number would continue to increase. Now a reasonable assumption is that by the end of 2022, the dream of “one center, one stop, and one standard model” implemented in more than 1000 clinics of MMC in China will have been realized.^7^ Aiming to establish a standardized diagnosis, treatment, and long‐term follow‐up platform for metabolic diseases, hospitals and clinics share the same structure in terms of facilities, layout, and databases, as well as the same daily operations. With the MMC clinics, patients can enjoy one‐stop care to get a series of comprehensive services from registration to detection to technological innovation.

Within 6 years, the MMC has dramatically changed the prevention and treatment mode all over China. There is still room for improvement and increase in level of care in the clinics. This outstanding breakthrough in medicine should be implemented throughout China in the coming years.

目前有超过5.3亿人受到全球糖尿病流行的影响。糖尿病在发展中国家尤其是一个问题。国际糖尿病联合会(IDF)2021年年鉴预测, 到2030年糖尿病患者数量将上升到6.43亿人, 其中四分之三的成人糖尿病患者生活在低收入和中等收入国家。在低收入和中等收入国家的大量糖尿病患者中, 60%以上在亚洲, 约三分之一在中国。中国最新的流行病学研究显示, 近11%的人口被诊断为糖尿病, 还有很大一部分人尚未确诊, 表明中国经历了世界上最显著的患病率增长之一。

如果同时患有糖尿病, 那么慢性肾病, 心血管疾病, 卒中, 感染和某些癌症等慢性疾病与死亡率较高有关。在1993年至2007年期间, 中国与糖尿病相关的医疗费用从22亿元人民币飙升至2000亿元人民币, 到2030年预计将超过3600亿元人民币。糖尿病管理对中国的挑战被认为是世界上最重要的例子之一, 给医疗系统带来了巨大的负担。

在中国, 高血压很普遍, 而且患病率还在上升。在35‐75岁的成年人中, 控制率低在所有亚组中都是如此普遍, 因此迫切需要一项基础广泛的全球战略, 如更多的预防, 更好的筛查和具有成本效益的治疗。中国居民对高血压和血脂异常的知晓率, 治疗率和控制率均明显低于美国。

由于老龄化人口的增长, 缺乏糖尿病教育人员, 以及缺乏结构化的医疗保健服务, 中国人心血管危险因素的患病率很高。社会孤立, 社会差异和获得护理的不平等性, 以及超重和肥胖的高流行率以及较低的社会经济地位, 导致人们对糖尿病缺乏认识。

为了提高中国慢性病的管理水平, 有效预防危险因素至关重要。基于年龄, 性别, 腰围, 体重指数, 收缩压和糖尿病家族史等主要预测因素, 研究人员开发了中国糖尿病风险评分, 以帮助识别高危个体。鉴于中国高危人群数量众多, 加强体力活动, 改善饮食的综合行为干预是预防糖尿病的关键。糖尿病护士, 营养师, 药剂师和医生应该更多地参与患者护理, 无论是面对面沟通还是电子医疗。

由于中国不同地区的经济, 文化发展和医疗服务标准存在巨大差异, 推荐的糖尿病指南与现实世界的糖尿病医疗保健之间存在差距。为了应对这些挑战, “国家代谢管理中心”(MMC)于2016年成立, 作为一个可在中国各地复制的试点和标准系统。

MMC旨在推出基于互联网健康信息平台的代谢性疾病社区管理新模式。此外, MMC有助于提高患者的治疗依从性和有效性, 从而造福于患者, 医生和医务人员。MMC专有的电子医疗数据库支持对糖尿病的流行病学, 预防, 诊断和治疗进行动态大数据分析。此外, 全国性的标准化护理系统还为糖尿病治疗和预防的前瞻性干预研究提供了一个协作平台。来源于MMC的大量数据和证据, 有助于实现长期最佳的健康结局, 从而在中国各地建立可复制的标准医护模式, 为未来医疗政策的制定和改进奠定基础。MMC的目标是将中国的糖尿病发病率降低1%, 糖尿病相关并发症的发生率降低10%。

MMC还开发了基于人工智能技术的医疗工具, 以促进其对糖尿病的筛查, 诊断和治疗。此外, MMC致力于在医院内外建立高效的诊断和治疗以及全面的疾病管理。

预计到2022年底, 在中国1000多家MMC诊所实施的“一中心, 一站式, 一标准模式”的构想可以实现…

在6年的时间里, MMC极大地改变了中国各地的预防和治疗模式。MMC的治疗水平仍有改进和提高的空间, 这一医学上的杰出突破应该在未来几年内在中国各地实施。
